# Patient Characteristics Associated with Definitive Diagnosis of Metastatic Pancreatic Cancer in Those Initially Diagnosed with Cancer of Unknown Primary

**DOI:** 10.21203/rs.3.rs-2833560/v1

**Published:** 2023-04-21

**Authors:** Larissa White, Julie Smith Gagen, Leslie Elliott, Minggen Lu

**Affiliations:** Kaiser Permanente Colorado Institute for Health Research; University of Nevada Reno; University of Nevada Reno; University of Nevada Reno

**Keywords:** Neoplasms, Unknown Primary, Pancreatic Neoplasms, Medicare, Health Services

## Abstract

**Purpose.:**

Cancer of unknown primary (CUP) is the fourth most common cause of cancer mortality in the U.S. Median survival after CUP diagnosis is 3–4 months. As CUP and metastatic pancreatic cancer (PC) are comparable in prevalence and survival, PC diagnosis is a useful endpoint to assess patient characteristics associated with definitive diagnosis in older patients who initially present with CUP.

**Methods.:**

This study used 2010–2015 SEER-Medicare data. Logistic regression models compared patient characteristics who received definitive diagnosis in two subsets: CUP-PC and PC only.

**Results.:**

Approximately 26% of patients who received a definitive diagnosis of metastatic pancreatic cancer started with an initial diagnosis of CUP (n=17,565). The odds of definitive diagnosis in CUP-PC were lower for those with a comorbidity score of 0 (OR 0.85 [0.79, 0.91]) and epithelial/unspecified histology (OR 0.76 [0.71, 0.82]). The odds of definitive diagnosis in CUP-PC were higher for patients of Other race (OR 1.27 [1.13, 1.43]) compared to White patients.

**Conclusion.:**

Definitive diagnosis of CUP-PC was favorable in patients in the Other race category with fewer or no comorbidities. Unfavorable characteristics included older patients and those with epithelial/unspecified histology. Future studies will focus on patterns of care and survival in patients with CUP-PC.

## 1. Purpose

Cancer of unknown primary (CUP), also known as occult cancer, accounts for approximately 3–5% of all cancers and is the fourth most common cause of mortality due to cancer in the U.S. [1–2]. CUP is defined as a case of metastatic cancer where the origination site cannot be determined [1]. Median survival after CUP diagnosis is approximately 3–4 months, with less than 25% of patients alive after one year [1–2]. Patients with CUP receive significantly less treatment yet use more health services when compared to patients with metastatic cancer of a known site [3]. Relatedly, pancreatic cancer accounts for approximately 3% of all cancers and is the third most common cause of mortality due to cancer in the U.S. [4–5]. The most critical prognostic factor for pancreatic cancer is stage at diagnosis [6]. Median survival time after stage 1 and stage 2 pancreatic cancer diagnosis is 4 months, with stage 3 and stage 4 pancreatic cancer decreasing survival time to 2–3 months [7]. Approximately 53% of pancreatic cancer patients are diagnosed after metastasis has occurred [4]. Since CUP and metastatic pancreatic cancer are comparable in prevalence and survival outcome, definitive pancreatic cancer diagnosis is a useful endpoint to assess patient characteristics associated with definitive diagnosis in older patients who initially present with CUP. Definitive diagnosis is crucial to the prognosis of patients diagnosed with CUP, but studies examining favorable characteristics of this outcome remain under-investigated [8–10]. Therefore, we sought to build upon our previous research [10] to examine which patient characteristics are associated with definitive diagnosis of metastatic pancreatic cancer in older patients who initially present with CUP.

## 2. Methods

This study uses 2010–2015 Surveillance, Epidemiology, and End Results (SEER)-Medicare data, a national population-based cancer registry linked to Medicare claims. The cohort consisted of patients identified in the SEER dataset diagnosed with CUP, International Classification of Diseases for Oncology, Third Edition (ICD-O-3) codes C80.9 and those diagnosed with stage 3 and stage 4 pancreatic cancer (ICD-O-3 codes C250-C259), between January 1, 2010 and December 31, 2015 [11]. Initial CUP diagnosis was defined by the date of the first biopsy or date of ICD-O-3 diagnosis, whichever came first. Patients had to be continuously enrolled in Medicare fee-for-service (both Part A and B) beginning 1 year prior to diagnosis through the observation period. Only the first reported primary cancer for each patient was included, that is, this was the first time the patients had been diagnosed with any type of cancer. Exclusion criteria were used to maximize patients whose claims data were complete: patients were excluded if enrolled in Medicare due to chronic disability, as well as those diagnosed only on a death certificate, at autopsy, or in a nursing home as their care was likely dissimilar to other patients. Only claims paid by Medicare were included so as to avoid erroneous billing codes. The final cohort consisted of 68,146 patients, of which 17,565 were initially diagnosed with CUP prior to a pancreatic cancer diagnosis.

### 2.1 Patient Characteristics.

Patient characteristics included gender, age in four groups (65 or younger, 66–74, 75–84, 85 and older), race (White, Black, and Other (which included Asian, American Indian/Alaska Native, Native Hawaiian/Other Pacific Islander, and Multi-racial)), ethnicity (Latino or Non-Latino), area of residence (rural or urban), and histology of the primary tumor (adenocarcinoma, squamous cell carcinoma, epithelial/unspecified, and neuroendocrine). Comorbidity was assessed utilizing the Klabunde adaptation of the Charlson comorbidity score [12].

### 2.2 Definitive Diagnosis.

Odd ratios (OR) and 95% confidence intervals (CI) were calculated using two binary logistic regression models to analyze the patient characteristics of who received definitive diagnosis between the CUP-Pancreas group (those with an initial diagnosis of CUP who eventually received a stage 3 or 4 pancreatic cancer diagnosis) and the Pancreas group (those diagnosed with stage 3 or 4 pancreatic cancer only). All analyses were conducted using SAS, version 9.4 (Cary, NC).

## 3. Results

Approximately 26% of patients who received a definitive diagnosis of stage 3/4 pancreatic cancer started with an initial diagnosis of CUP (n = 17,565). Of these cases, 53.4% were female, 37.3% were between the ages of 75–84, 81.6% were White, 92.3% were non-Latino, 59.1% lived in an urban area, 42.6% had a Charlson comorbidity score of 2 or higher, and 60.7% were histologically confirmed as adenocarcinoma (Table 1). Of the cases diagnosed only with stage 3/4 pancreatic cancer, characteristics were generally similar to those initially diagnosed with CUP, however, 29.9% were between the ages of 75–84 and 31.9% had a Charlson comorbidity score of 2 or higher.

**Figure 1:**
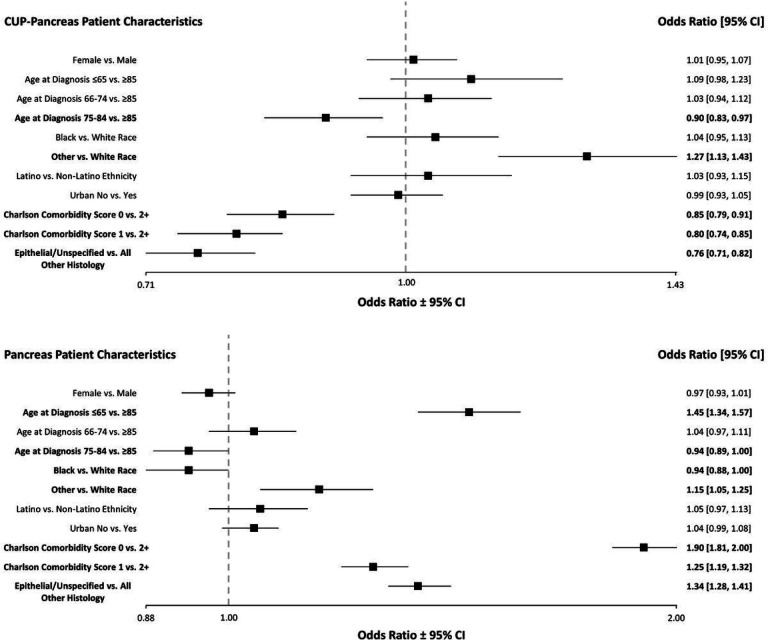
Odds ratios of definitive pancreatic cancer diagnosis in those initially diagnosed with CUP (N=17,565) compared to those diagnosed only with pancreatic cancer (N=50,581) by patient characteristics, SEER-Medicare, 2010–2015

The odds of definitive pancreatic cancer diagnosis in patients initially diagnosed with CUP (Fig. 1) were lower for patients between the ages of 75–84 (OR 0.90 [0.83, 0.97]) compared to patients 85 years or older; lower for those with a Charlson comorbidity score of 0 (OR 0.85 [0.79, 0.91]) or 1 (OR 0.80 [0.74, 0.85]) compared to a score of 2 or higher; and lower for epithelial/unspecified histology compared to all other histology types (OR 0.76 [0.71, 0.82]). The odds of definitive pancreatic cancer diagnosis in patients initially diagnosed with CUP was higher for patients of Other race (OR 1.27 [1.13, 1.43]) compared to White patients.

The odds of definitive diagnosis in patients with pancreatic cancer only was lower for patients between the ages of 75–84 (OR 0.94 [0.89, 1.00]) compared to patients 85 years or older and lower for Black patients (OR 0.94 [0.88, 1.00]) compared to White patients (Fig. 1). The odds of definitive diagnosis in patients only with pancreatic cancer were higher for patients 65 years or younger (OR 1.45 [1.34, 1.57]) compared to patients 85 years or older; higher for patients of Other race (OR 1.15 [1.05, 1.25]) compared to White patients; higher for those with a Charlson comorbidity score of 0 (OR 1.90 [1.81, 2.00]) or 1 (OR 1.25 [1.19, 1.32]) compared to a score of 2 or higher; and higher for epithelial/unspecified histology compared to all other histology types (OR 1.34 [1.28, 1.41]).

## 4. Discussion

To our knowledge, this is the first population-based study focusing on metastatic pancreatic cancer in patients initially diagnosed with CUP. This study examined patient characteristics associated with definitive diagnosis of metastatic pancreatic cancer in older patients who initially presented with CUP.

Definitive diagnosis of stage 3 or stage 4 pancreatic cancer in patients who initially presented with CUP was favorable in patients in the Other race category with fewer or no comorbidities. Unfavorable characteristics for definitive diagnosis included patients in older age groups and histology confirmed as epithelial/unspecified. Patients with comorbidities may receive health services more often than patients without comorbidities, thus are more likely to come in contact with the health care system [13]. However, older patients with comorbidities may be unable to complete the diagnostic workup necessary to make a definitive diagnosis [14]. Unfavorable characteristics for definitive diagnosis of CUP to a specified primary site including older age, epithelial/unspecified histology, and higher comorbid burden of disease correspond with current scientific literature on CUP patterns of care, namely population-based studies focusing on patient characteristics and healthcare utilization [3, 15–16], adherence and diagnostic guidelines [10], and risk factors and clinical management [17–18].

In patients diagnosed with stage 3 or stage 4 pancreatic cancer only, definitive diagnosis was similar to CUP patients by race, however, this subpopulation was younger and had fewer comorbidities overall. Furthermore, the comorbidity score and whether histology was epithelial/unspecified were not barriers to definitive diagnosis for the pancreatic cancer only group, suggesting there are imbalances in delivery of care compared to patients initially diagnosed with CUP. This is likely due to (a) the complexity of identifying the primary tumor site in CUP, whereas in identification of pancreatic cancer, the clinician at least has a point from which to begin a well-informed diagnostic process; and (b) poor performance status of the patient with CUP, a potential confounder this study could not account for.

These findings further elucidate the health disparities evident in CUP and pancreatic cancer diagnoses. Scientific literature on cancer health disparities report higher incidence of metastatic pancreatic cancer among Black and Latino patients, as well as lower occurrence of treatment (primarily surgical intervention), poor access to quality health care, and higher rates of overall morbidity and mortality [19–21]. An area of future research should focus on the patterns of care associated with race, ethnicity, and social determinants of health (to include socioeconomic status) in patients diagnosed with CUP and pancreatic cancer.

While SEER-Medicare data provided a robust sample size, there are limitations in this study. Furthermore, our study population was limited to patients 65 years and older and did not include patients with private insurance coverage. However, the age range of an average patient with CUP is 80 years or older and the vast majority of patients 65 years and older are insured through Medicare [22]. This study only investigated patients with a final metastatic pancreatic cancer diagnosis. Further research of other CUP-primary site cancers, for example ovarian and lung cancers, would be beneficial to the growing scientific literature on the diagnostic complexity of CUP. It is also important to note clinicians may need to report a definitive diagnosis to justify treatment for insurance claims. Claims data for administrative and billing purposes might be inaccurate from a biological or clinical disease perspective.

## 5. Conclusion

This brief report provides an initial profile of favorable and unfavorable patient characteristics associated with the definitive diagnosis of metastatic pancreatic cancer in patients who initially presented with CUP based on a large and representative population-based cohort. Future studies will focus on patterns of care and survival outcomes in patients who initially present with CUP.

## Figures and Tables

**Table 1:** Descriptive characteristics of definitive pancreatic cancer diagnosis in those initially diagnosed with CUP compared to those diagnosed with pancreatic cancer only, SEER-Medicare, 2010–2015

Demographic Variable		
	CUP-Pancreas (n = 17,565)	Pancreas (n = 50,581)
Gender		
Female	9,387 (53.4%)	25,897 (51.2%)
Male	8,178 (46.6%)	24,684 (48.8%)
Age at Diagnosis		
<= 65	2,025 (11.5%)	12,038 (23.8%)
66–74	6,031 (34.3%)	16,237 (32.1%)
75–84	6,553 (37.3%)	15,123 (29.9%)
85+	2,956 (16.9%)	7,183 (14.2%)
Race		
White	14,329 (81.6%)	41,122 (81.3%)
Black	2,123 (12.1%)	5,564 (11.0%)
Other	1,113 (6.3%)	3,895 (7.7%)
Ethnicity		
Non-Latino	16,212 (92.3%)	46,180 (91.3%)
Latino	1,353 (7.7%)	4,401 (8.7%)
Urban		
Yes	10,379 (59.1%)	30,602 (60.5%)
No	7,186 (40.9%)	19,979 (39.5%)
Charlson Comorbidity Score		
0	5,337 (30.4%)	21,649 (42.8%)
1	4,748 (27.0%)	12,797 (25.3%)
2+	7,480 (42.6%)	16,135 (31.9%)
Histology		
Adenocarcinoma	10,661 (60.7%)	29,843 (59.0%)
Epithelial/Unspecified	3,627 (20.6%)	11,077 (21.9%)
Neuroendocrine	3,070 (17.5%)	9,105 (18.0%)
Squamous Cell Carcinoma	207 (1.2%)	556 (1.1%)

## Data Availability

The data that support the findings of this study are available from National Cancer Institute but restrictions apply to the availability of these data, which were used under contract for the current study, and so are not publicly available without formal request and application to the National Cancer Institute.
